# Unprecedented Diversity of Lactococcal Group 936 Bacteriophages Revealed by Amplicon Sequencing of the Portal Protein Gene

**DOI:** 10.3390/v11050443

**Published:** 2019-05-16

**Authors:** Cyril Alexander Frantzen, Helge Holo

**Affiliations:** 1 ACD Pharma AS, Karl Johans gate 16, 0154 Oslo, Norway; cyril.frantzen@acdpharma.com; 2 Laboratory of Microbial Gene Technology and Food Microbiology, Faculty of Chemistry, Biotechnology and Food Science, Norwegian University of Life Sciences, P.O.B. 5003, N-1432 Aas, Norway; 3 Tine SA, N-0187 Oslo, Norway

**Keywords:** Lactococcus, bacteriophage group 936, dairy, culture independent, phage diversity

## Abstract

*Lactococcus lactis* is one of the most important bacteria in dairy fermentations, being used in the production of cheese and buttermilk. The processes are vulnerable to phage attacks, and undefined mixtures of lactococcal strains are often used to reduce the risk of bacteriophage caused fermentation failure. Other preventive measures include culture rotation to prevent phage build-up and phage monitoring. Phage diversity, rather than quantity, is the largest threat to fermentations using undefined mixed starter cultures. We have developed a method for culture independent diversity analysis of lytic bacteriophages of the 936 group, the phages most commonly found in dairies. Using, as a target, a highly variable region of the portal protein gene, we demonstrate an unprecedented diversity and the presence of new 936 phages in samples taken from cheese production. The method should be useful to the dairy industry and starter culture manufacturers in their efforts to reduce phage problems.

## 1. Introduction

*Lactococcus lactis* is a key player in cheese production, and lactococcal bacteriophages can have detrimental effects in cheesemaking. Bacteriophage attacks can cause slow or insufficient milk acidification and reduced product quality, which is recognized after prolonged ripening. Phages attacking *L. lactis* have been classified into ten groups, but in modern dairies only members of the three groups 936, C2 and P335 are common [1]. Among them, 936 like phages are most often found and are considered the most problematic [2,3,4,5]. In samples taken from Norwegian dairies, 936 phages were found in bulk starter and whey samples [2]. Moreover, using quantitative PCR, the levels were estimated to be 10^8^–10^10^ plaque forming units per ml. No P335 or C2 phages were found in the samples. However, despite the high phage numbers, no noticeable fermentation failure or cheese quality effects were observed in most cases.

Lactococcal phages can be highly strain specific [5,6]. Consequently, mixtures of strains are commonly used in cheese production, making the process robust against phage attacks [7]. The starter cultures for Dutch type cheeses contain undefined strain mixtures of *Lactococcus lactis* ssp. *lactis*, *L. lactis* ssp. *cremoris*, *L. lactis* ssp. *lactis* biovar *diacetylactis* and *Leuconostoc* spp. However, critical phage attacks do occur even when mixed starter cultures are used. In a study with daily phage monitoring, slow acidification was found to coincide with an increase in phage diversity [2].

Along with good sanitation procedures, an important measure to reduce phage problems is culture rotation [8]. This involves alternating the use of two or more cultures with different strain composition. Because of the narrow host spectra of most phages, the cultures will not propagate each other’s phages. Culture rotation is thus likely to prevent build-up of phages in the dairy plant. However, changes to another starter culture may be at the cost of reduced product quality, such as undesirable taste and texture. Phage monitoring is also paramount as a supportive measure to control phage problems in the dairy industry. Traditional culture-based techniques can be used to record gross acidification rate defects. Phages can be quantified by plaque testing, but this is laborious and for an undefined mixed culture such testing would require the isolation of pure host strains for all phages. Such strain isolation itself is likely to be difficult as not all strains are isolated by plating (or culturable) on standard laboratory media [6,9].

DNA based methods, such as qPCR, are useful for phage quantification [10,11]. With qPCR, phage gross compositions are quantified, but not the nuances that can be picked up by plaque tests; thus, PCR cannot register the influence of a phage on strain balance in the fermentation. The finding that high phage diversity rather than quantity is associated with fermentation failure using mixed starters [2] underpins the need for high resolution phage analyses.

Being culture independent and with its great sequencing depth, next-generation sequencing has revolutionized the study of biological diversity. It is widely used in microbiome analyses, and also in the analysis of viromes, albeit more difficultly [12]. Targeted amplicon sequencing is the method most often used in microbiome analyses. This method focuses on a diversity of a specific marker, like 16S rDNA. In this work, we have developed a method based on targeted amplicon sequencing to identify and quantify the diversity of 936 phages. Using this method, we show an unprecedented diversity of such phages in samples from cheese production.

## 2. Materials and Methods

### 2.1. Cultivation and Isolation of Bacteriophages

All bacteriophage isolates used in this study were isolated from Norwegian cheese production in a previous study [6], and phage propagation and cultivation was performed as described there.

### 2.2. Genome Sequencing, Assembly, and Annotation

Genomic DNA from bacteriophage isolates was extracted from 200 µL of high-titer (>10^7^ bacteriophages/mL) lysates using the DNeasy blood and tissue kit (Qiagen, Hilden, Germany). To remove bacterial DNA prior to column purification, the lysates were treated with 2U DNase I for 30 minutes at 37 °C which was inactivated by 50 mM EDTA and 1% SDS while opening the bacteriophage capsids with 3U of proteinase K for 30 min at 55 °C. The phages were identified as 936 phages by PCR [10]. DNA libraries were constructed using the Nextera XT DNA Sample Prep kit (Illumina, San Diego, CA, USA) according to the manufacturer’s instructions and sequenced on an Illumina MiSeq (Illumina, San Diego, CA, USA) platform using V3 chemistry. Raw sequences were adapter trimmed, quality filtered (Q > 20), de novo assembled using SPAdes V3.11.1 [13] and annotated using the Prokka v1.12 pipeline [14]. Contigs shorter than 1000 bp or with less than 5× coverage were removed from each assembly prior to gene annotation. In addition, 186 publically available complete 936 bacteriophage genomes were acquired from the NCBI genomes database. These genomes were all reannotated using the Prokka v1.12 pipeline.

### 2.3. Pan-/Core-Genomic Analyses

The protein coding sequences of all isolates were compared by an all-against-all approach and grouped into orthologous clusters using Roary [15]. The orthologous clusters were curated to exclude significantly divergent singletons, which are likely to be the result of erroneous assembly or annotation. A pan-genomic presence/absence matrix was constructed, including each gene cluster and each genome. Hierarchal single-linkage clustering analysis of this matrix was performed in R (http://www.r-project.org/) to construct a pangenome heatmap overview using the heatmap.2 function included in the Gplots package v2.16 [16] supplemented by the dendextend package v0.18.3 [17]. Core genes were included in a multi-locus multiple alignment scheme to determine the phylogenetic distances between genomes and to construct a Maximum Likelihood (ML) phylogenetic supertree using RAxML-ng v0.8.1b [18] and the sequence alignment metric functions in the DECIPHER v2.0 [19] and MASS v7.3-47 [20] packages in R. A suitable amplicon target was identified by screening the core genes for nucleotide sequence variation using the sequence alignment metric functions in the DECIPHER package v1.16.1 [21]. Genes without flanking consensus regions within a 500 bp variable region adequate for differentiation or which did not provide sufficient discrimination between lineages were discarded. Maximum Likelihood phylogenetic trees for the operational taxonomic units were constructed using RAxML-NG v0.8.1.b [18].

### 2.4. Relative Quantification of the Bacteriophage Diversity in Dairy Samples

The bacteriophage diversity analyses were performed in triplicates on DNA extracted from 200 μl of the supernatant from bulk-starter and whey samples from cheese production using the same procedure as for genomic DNA isolation. The portal protein gene fragment was amplified by PCR using the Kapa HiFi PCR kit (Kapa Biosystems, Wilmington, MA, USA) and annealing at 55 °C with primers Portal-471F (5′-GCTGGAACAAGGTAAATTGCGT-3′) and Portal-931R (5′-AGTCAATTAATTCTTTCAAAGTTGCAA-3′). Forward (5′-TCGTCGGCAGCGTCAGATGTGTATAAGAGACAG) and reverse (5′-GTCTCGTGGGCTCGGAGATGTGTATAAGAGACAG) Illumina adapter overhangs were added to the 5′ ends of the primers to enable Nextera XT DNA indexing of the PCR products. The libraries were sequenced on the Illumina MiSeq platform using V3 (2 × 300 bp) reagents. The resulting data were paired-end merged and quality filtered using PEAR v0.9.6 [22] and clustered using VSEARCH v2.4.3 [23] with error minimization from USEARCH v10.0.240 [24]. The amplicon data was clustered by a similarity threshold of 99.5%, corresponding to a nucleotide difference of two single-nucleotide polymorphisms. The resulting operational taxonomic unit (OTU) was matched against a local BLAST database produced using the bacteriophage genomes sequenced in this study as well as the bacteriophage genomes available on the NCBI database.

### 2.5. Accession Number(s)

The whole-genome project and OTUs have been deposited at DDBJ/ENA/GenBank under BioProject number PRJEB32164.

## 3. Results

**Figure 1:**
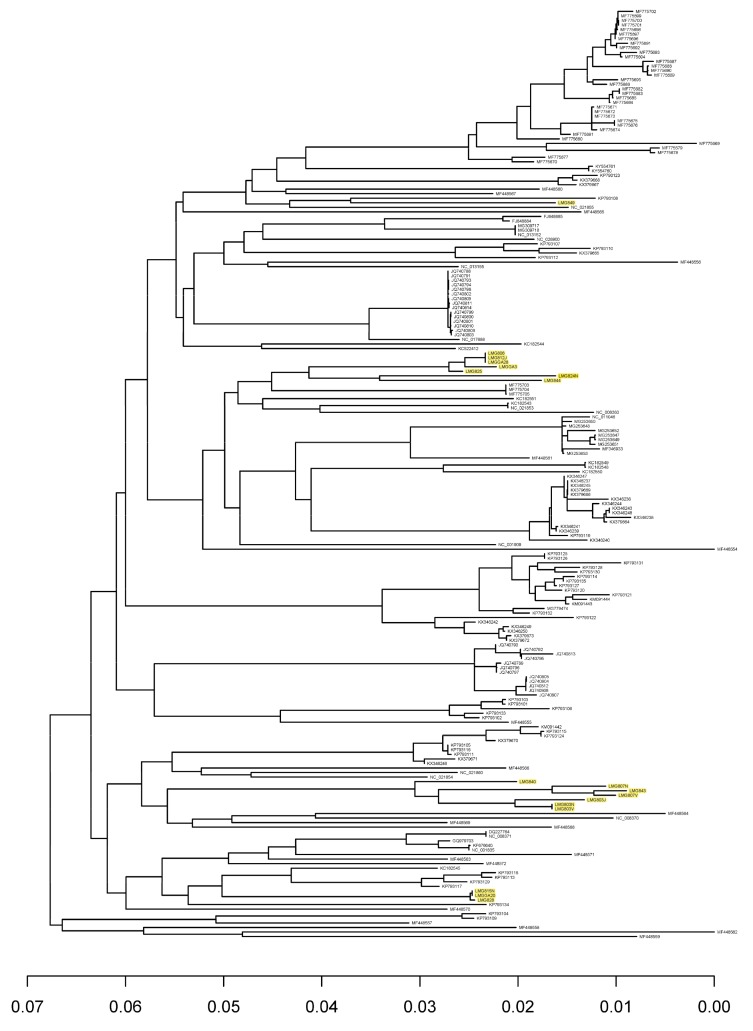
Phylogenetic tree of group 936 bacteriophages based on 29 core genes. Bacteriophages included in this study are given as “LMG” followed by three numbers. Bacteriophages acquired from the NCBI are presented by their accession numbers. Bacteriophages isolated in this work are highlighted.

Using strains isolated from cheese starter cultures, we isolated several bacteriophages from Norwegian cheese production plants employing undefined mixed starter cultures [6]. Several of those were sequenced, and eighteen new phage genome sequences were obtained. All of them were identified as 936 type phages by BLAST searches, and their genome sequences were different from the 186 genome sequences of 936 phages in the NCBI database. The genomes vary in size from 28,522 to 32,980 base pairs (bp) and encode between 50 and 62 protein-coding sequences (Table S1). A core genome was determined based on these sequences and the sequences in the database. The core genome contained 29 genes (Table S2), the same core genes as found by Murphy et al. [4] using a smaller dataset. The core genome phylogenetic tree is shown in Figure 1. As shown in the Figure most of our phages are found in two separate clusters, corresponding to whether they attack a *Lactococccus lactis* subsp. *lactis* or a *Lactococccus lactis* subsp. *cremoris* host. Phages described in [25,26] show the same host dependent distribution. The number of non-core genes in the phages isolated in this work varied between 21 and 33. Similar numbers have been found previously [4].

Core genes from these 204 genomes were searched for variable regions suitable for diversity analyses by targeted amplicon sequencing using the same approach as described previously [9].

The upper size limit for deep sequencing amplicon analyses on the Illumina platform is about 500 bp. The gene showing the largest sequence diversity over a 500 bp stretch was *rbp*, the gene encoding the receptor binding protein, with 121 sequence variants. However, as shown previously [25], no single primer set giving an amplicon suitable for Illumina sequencing and applicable to all the known phages could be identified within the gene. We identified a region within the portal protein gene that showed almost as much sequence variation as *rbp*, flanked by highly conserved sequences and thus suitable for targeted amplicon sequencing by the Illumina MiSeq sequencer. Among the 204 phage genome sequences examined there were 103 sequence variants in the portal protein gene.

**Figure 2:**
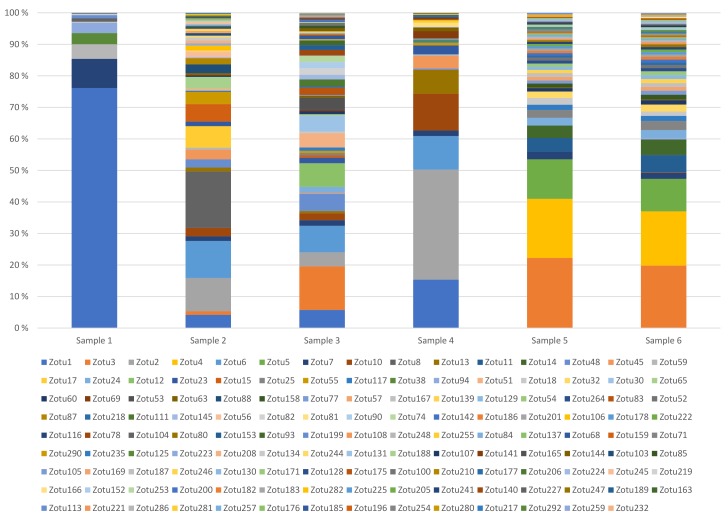
Bacteriophage diversity in six dairy samples using targeted amplicon sequencing of the portal protein gene. The samples were acquired from two separate dairy facilities. Samples 1–3 are from one location, while samples 4–6 are from a second location. Sample 5 and 6 are produced using the same starter culture batch.

We generated a primer set for amplification of the highly variable region of the portal protein gene. The primers were estimated to be able to amplify the region of 201 of the 204 phages studied, and from them produce 81 different sequence variants. The primers were used in targeted amplicon analyses of six dairy samples. As shown in Table 1 and Figure 2, all samples contained wide varieties of phages. Moreover, there was substantial variation between samples, except from samples 5 and 6. Samples 5 and 6 were taken from the same production at two different time points of the day.

Using a threshold of 99.5% and a conservative cutoff of a minimum 100 reads per operational taxonomic unit (OTU) we identified 134 OTUs. An even higher number, 151 OTUs, could be discerned by only filtering out OTUs with abundances of less than the 0.005% of sequences as recommended by Bokulich et al. [27]. Our results are based on analyses using 100,000 to 200,000 reads per sample. With this sequencing depth the method and the filtering settings described above the method can quantify OTUs differing by 4 logs in relative abundance.

No OTU showed less than 91% sequence identity to any of the other OTUs in the dairy samples, and all encoded an uninterrupted reading frame. Sequence comparison with the NCBI database showed them all to be very similar (minimum 88% identity), but not identical, to phage 936 portal protein sequences in the NCBI database. Eight of the 134 OTUs shared 100% sequence identity with phages sequenced in this work.

Samples 1, 2 and 3 were from one dairy plant, and the rest from another. Only 30 of the OTUs were represented in both dairy plants, 54 OTUs were specific for dairy 1 and 50 OTUs were specific for dairy 2. Only one OTU, OTU 7, was found in all samples. Based on the OTU sequences, we created a phylogenetic tree, which shows the relationships between OTUs detected in the two dairy plants (Figure S1). OTUs representing the isolated phages are also included, and the clustering of these OTUs correlates well with their clustering in Figure 1. As shown in Figure S1, several of the OTU clusters were only represented in one of the two dairies. We also made a phylogenetic tree including the unique OTUs from the 936 phage genomes in the NCBI database (Figure S2). Two new deep branching lineages are seen in the tree, but most of the OTUs cluster with OTUs found in the dairy samples.

## 4. Discussion

Infection by 936 phages is a never-ending problem in the dairy industry, and phage monitoring is necessary in troubleshooting and in the work to control phage attack. Phage richness is an important parameter related to fermentation failures with mixed starter cultures [2], and it is instrumental to assay diversity and not only abundance in phage monitoring.

Viruses do not have a universal phylogenetic marker analogous to the bacterial 16S rRNA gene for diversity analyses. However, the late-expressed genes are highly conserved in 936 phages. Among them, the portal protein gene was the best suited to fulfill the criterion of a narrow region of high sequence variability flanked by highly conserved sequences. The primers constructed are estimated to recognize 99% of the known fully genome sequenced 936 phages studied here, and their applicability was proven using dairy samples. The analyses of bulk starters and wheys from Norwegian cheese plants revealed a huge number of variants of 936 phages. Nearly all of the OTUs we discovered in the dairy samples represented new bacteriophages. We do not know the hosts of these phages, except that they are found in the starter cultures. The phage richness found in the dairy samples, 134 OTUs, is much higher than the 66 OTUs that represent the fully sequenced 936 phages genomes in the NCBI database.

Our samples from two dairies also showed great variations between sampling days and between the plants themselves. Apparently, the two dairy plants have their own phage floras. This is in agreement with findings by Murphy et al. [4], who noticed a correlation between 936 genome sequence and geographic site of isolation. The phylogenetic analysis of the OTUs revealed clusters that appeared to be specific for a dairy plant. However, most of this apparent specificity was lost when the data from the genome sequences were included.

In a previous work we were able to identify up to 31 lactococcal bacterial lineages (OTUs) in a mixed starter culture [9]. The much higher richness of phage OTUs clearly indicates that many of the strains were infected by more than one phage, possibly at the same time, allowing for genetic recombination between different 936 phages [26]. In the study of phage evolution in a dairy over 29 years, Kupczok et al. [26], found no evidence for genetic recombination in the portal protein gene. The gene is essential for viral replication and highly conserved, yet it shows great sequence variation. These features make it a good candidate as a marker gene for phylogenetic analyses (a molecular chronometer). Portal protein phylogeny has been established from highly diverse members of the *Caudovirales* [28,29].

A key question about phages discovered by PCR is what hosts they can infect. The lactococcal cell wall polysaccharide (CWPS) has been identified as a surface receptor for the 936 group phages [30,31,32,33,34]. Moreover, the 936 phage receptor binding protein is involved in CWPS binding and a correlation has been found between the phage’s *rbp* sequence and the sequence of the CWS gene cluster of sensitive hosts [4,32]. Like for the portal protein gene, there was no evidence for recombination events in *rbp* evolution [26]. Since the portal protein gene and *rbp* are genetically linked, the amplicon analysis might be useful in prediction a phage’s *rbp* [4] and hence in predicting a host spectrum for the phage. However, exceptions are found. Most notably, among our sequenced phages, LMG8825 and LMGGA3 had identical *rbp* but highly different portal protein genes with only 95% identity. This indicates that the phages have evolved from a common ancestor in a process involving horizontal gene transfer. Moreover, the two phages show slightly different host specificities, showing that *rbp* alone is not the only factor determining host specificity.

Culture independent methods have provided new insight into biological diversity. We have disclosed the presence of a hitherto unknown large number of different phages in dairy samples, but we do not know their hosts or whether they can be propagated at all by standard laboratory techniques. Further DNA sequencing can provide more insight into these phages. Their abundance demonstrates an important impact on culture composition, and possibly effects on product quality. For the dairy industry, the new method offers a new, in-development tool to control phage and develop robust starter cultures.

## 5. Conclusions

We describe a culture independent method for diversity analysis of 936 phages. We show its usefulness on dairy samples and suggest it as tool in phage monitoring. The results obtained greatly expand our knowledge about the diversity of 936 phages.

## Figures and Tables

**Table 1:** Diversity of 936 phages in dairy samples from two Norwegian dairy plants; OTU, operational taxonomic unit.

Dairy Sample	1	2	3	4	5	6
Dairy Plant	1	1	1	2	2	2
Number of OTUs	11	46	55	26	54	59

## Supplementary Materials

The following are available online at https://www.mdpi.com/1999-4915/11/5/443/s1, Figure S1: Phylogeny of OTUs from Norwegian cheese production. OTUs inferred from genome sequences of phages isolated in this work are included. OTUs only found in dairy 1 is shown in green, OTUs only found in dairy 2 is shown in blue, OTUs found in both dairy plants are shown in pink. OTUs representing phages isolated in this work are shown in black., Figure S2: Phylogeny of OTUs from Norwegian cheese production and published 936 phage genome sequences and OTUs inferred from genome sequences of 936 phages. OTUs only found in dairy 1 is shown in green, OTUs only found in dairy 2 is shown in blue, OTUs found in both dairy plants are shown in pink. OTUs representing phages isolated in this work are shown in black. OTUs representing published phage genome sequences (66 unique OTUs) are shown in grey. The published sequences are represented by their accession numbers. Table S1: Genomes of bacteriophages isolated in this study, Table S2: Core genome of group 936 bacteriophages. The ORFs are number according to numbering in bacteriophage sk1.
