# Supplementary material for: Unprecedented Diversity of Lactococcal Group 936 Bacteriophages Revealed by Amplicon Sequencing of the Portal Protein Gene
Source: Viruses. 2019 May 16;11(5):443. doi: 10.3390/v11050443 (PMC6563314; doi:10.3390/v11050443)
Supplement: Supplementary file 1 [file viruses-11-00443-s001.zip › viruses-496482-proofread back suppl/FigureS1.pdf]

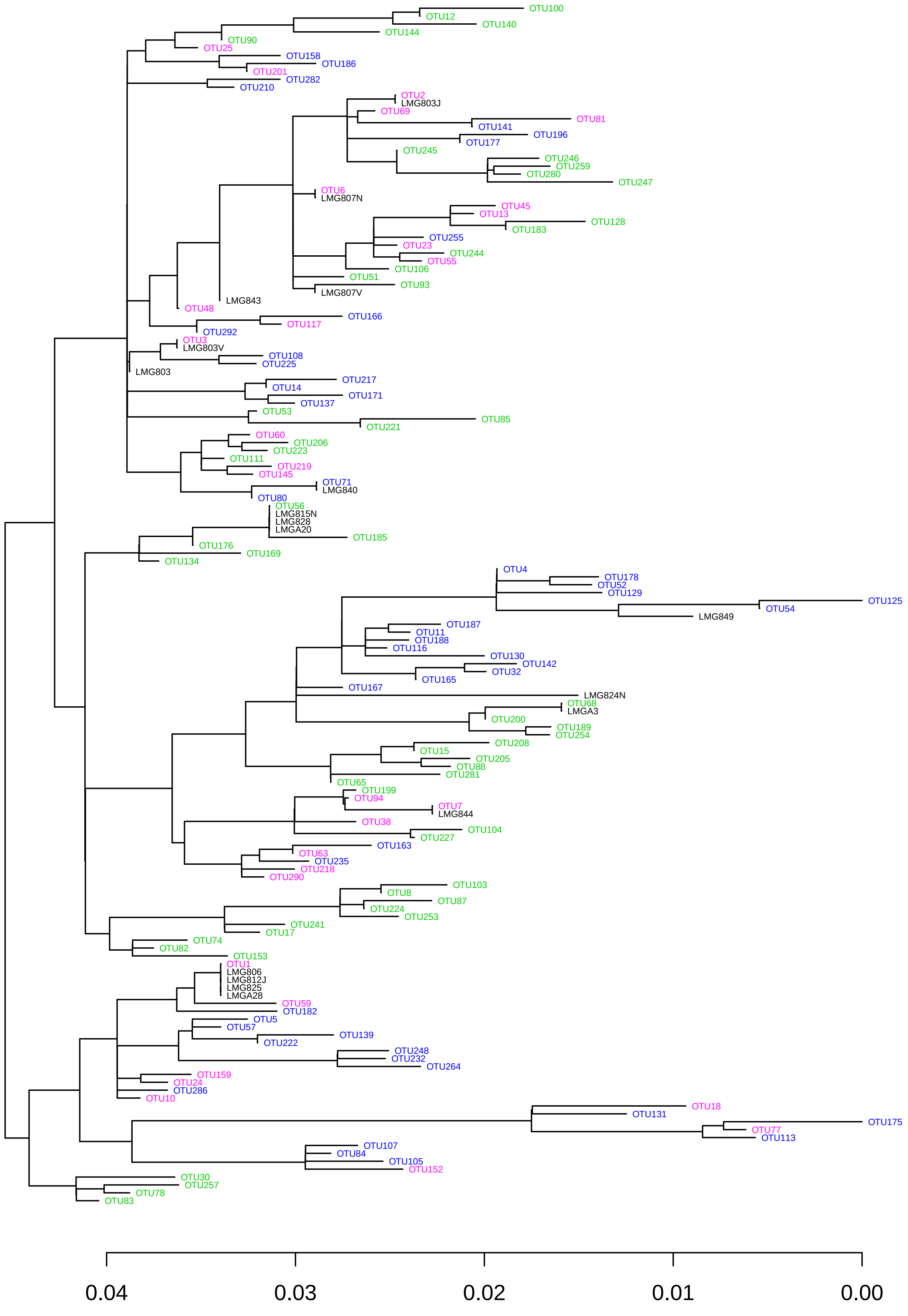
OTU100
OTU12
OTU140
OTU144
OTU90
OTU25
OTU158
OTU186
OTU201
OTU282
OTU210
OTU2
LMG803J
OTU69
OTU81
OTU141
OTU196
OTU177
OTU245
OTU246
OTU259
OTU280
OTU247
OTU6
LMG807N
OTU45
OTU13
OTU128
OTU183
OTU255
OTU23
OTU244
OTU55
OTU106
OTU51
OTU93
LMG807V
LMG843
OTU48
OTU166
OTU117
OTU292
OTU3
LMG803V
OTU108
OTU225
LMG803
OTU217
OTU14
OTU171
OTU137
OTU53
OTU85
OTU221
OTU60
OTU206
OTU223
OTU111
OTU219
OTU145
OTU71
LMG840
OTU80
OTU56
LMG815N
LMG828
LMGA20
OTU185
OTU176
OTU169
OTU134
OTU4
OTU178
OTU52
OTU129
OTU125
OTU54
LMG849
OTU187
OTU11
OTU188
OTU116
OTU130
OTU142
OTU32
OTU165
OTU167
LMG824N
OTU68
LMGA3
OTU200
OTU189
OTU254
OTU208
OTU15
OTU205
OTU88
OTU281
OTU65
OTU199
OTU94
OTU7
LMG844
OTU38
OTU104
OTU227
OTU163
OTU63
OTU235
OTU218
OTU290
OTU103
OTU8
OTU87
OTU224
OTU253
OTU241
OTU17
OTU74
OTU82
OTU153
OTU1
LMG806
LMG812J
LMG825
LMGA28
OTU59
OTU182
OTU5
OTU57
OTU139
OTU222
OTU248
OTU232
OTU264
OTU159
OTU24
OTU286
OTU10
OTU18
OTU131
OTU175
OTU77
OTU113
OTU107
OTU84
OTU105
OTU152
OTU30
OTU257
OTU78
OTU83
0.04
0.03
0.02
0.01
0.00

Figure S1

Phylogeny of OTUs from Norwegian cheese production. OTUs inferred from genome sequences of phages isolated in this work are included. OTUs only found in dairy 1 is shown in green, OTUs only found in dairy 2 is shown in blue, OTUs found in both dairy plants are shown in pink. OTUs representing phages isolated in this work are shown in black.
