# Supplementary material for: Unprecedented Diversity of Lactococcal Group 936 Bacteriophages Revealed by Amplicon Sequencing of the Portal Protein Gene
Source: Viruses. 2019 May 16;11(5):443. doi: 10.3390/v11050443 (PMC6563314; doi:10.3390/v11050443)
Supplement: Supplementary file 1 [file viruses-11-00443-s001.zip › viruses-496482-proofread back suppl/FigureS2.pdf]

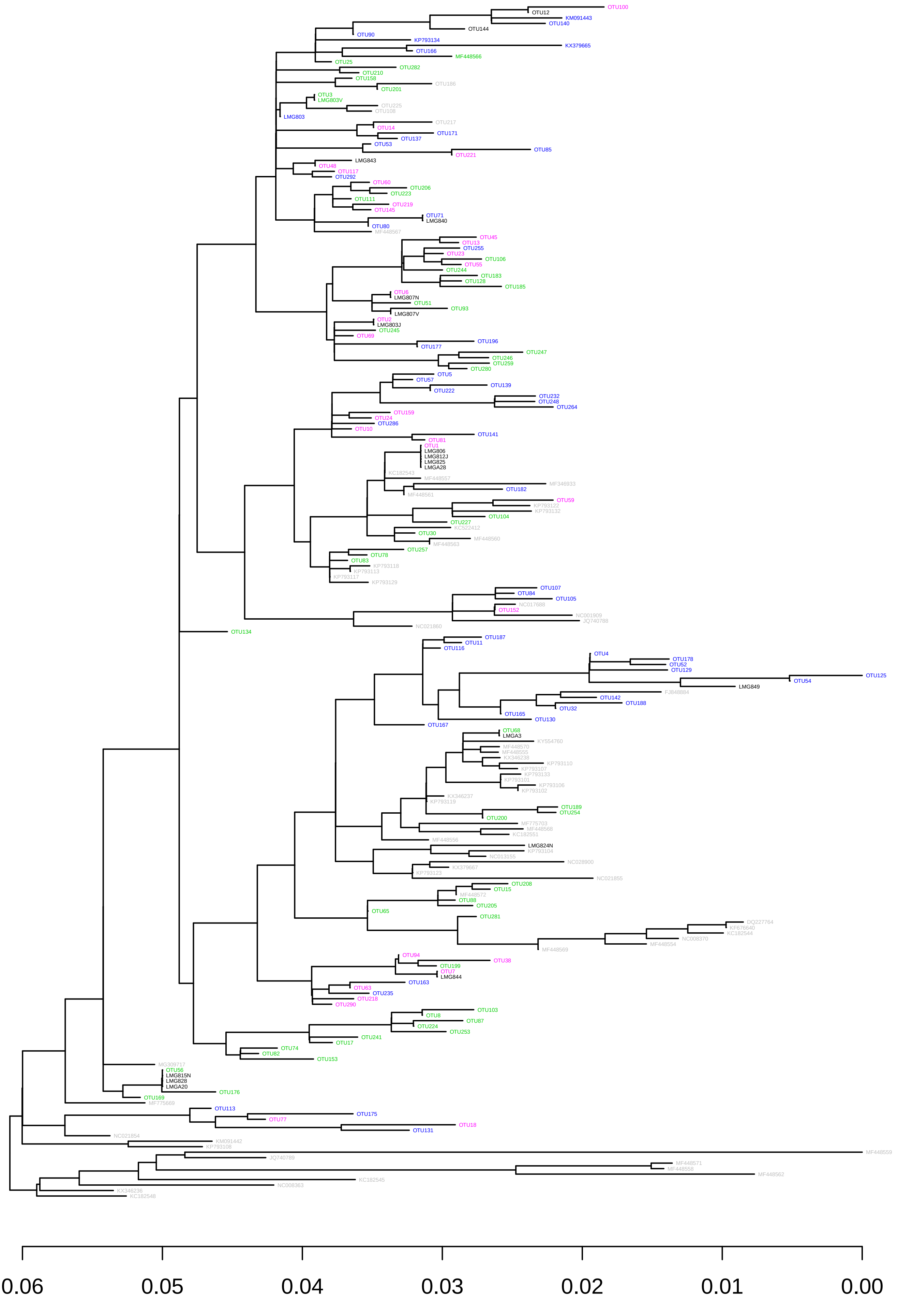
OTU100
OTU12
KM091443
OTU140
OTU144
OTU90
KP793134
OTU166
KX379665
MF448566
OTU25
OTU282
OTU210
OTU158
OTU186
OTU201
OTU3
LMG803V
OTU225
OTU108
LMG803
OTU217
OTU14
OTU171
OTU137
OTU53
OTU85
OTU221
LMG843
OTU48
OTU117
OTU292
OTU60
OTU206
OTU223
OTU111
OTU219
OTU145
OTU71
LMG840
OTU80
MF448567
OTU45
OTU13
OTU255
OTU23
OTU106
OTU55
OTU244
OTU183
OTU128
OTU185
OTU6
LMG807N
OTU51
OTU93
LMG807V
OTU2
LMG803J
OTU245
OTU69
OTU196
OTU177
OTU247
OTU246
OTU259
OTU280
OTU5
OTU57
OTU139
OTU222
OTU232
OTU248
OTU264
OTU159
OTU24
OTU286
OTU10
OTU141
OTU81
OTU1
LMG806
LMG812J
LMG825
LMGA28
KC182543
MF448557
MF346933
OTU182
MF448561
OTU59
KP793122
KP793132
OTU104
OTU227
KC522412
OTU30
MF448560
MF448563
OTU257
OTU78
OTU83
KP793118
KP793113
KP793117
KP793129
OTU107
OTU84
OTU105
NC017688
OTU152
NC001909
JQ740788
NC021860
OTU134
OTU187
OTU11
OTU116
OTU4
OTU178
OTU52
OTU129
OTU125
OTU54
LMG849
FJ848884
OTU142
OTU188
OTU32
OTU165
OTU130
OTU167
OTU68
LMGA3
KY554760
MF448570
MF448555
KX346238
KP793110
KP793107
KP793133
KP793101
KP793106
KP793102
KX346237
KP793119
OTU189
OTU254
OTU200
MF775703
MF448568
KC182551
MF448556
LMG824N
KP793104
NC013155
NC028900
KX379667
KP793123
NC021855
OTU208
OTU15
MF448572
OTU88
OTU205
OTU65
OTU281
DQ227764
KF676640
KC182544
NC008370
MF448554
MF448569
OTU94
OTU38
OTU199
OTU7
LMG844
OTU163
OTU63
OTU235
OTU218
OTU290
OTU103
OTU8
OTU87
OTU224
OTU253
OTU241
OTU17
OTU74
OTU82
OTU153
MG309717
OTU56
LMG815N
LMG828
LMGA20
OTU176
OTU169
MF775669
OTU113
OTU175
OTU77
OTU18
OTU131
NC021854
KM091442
KP793108
MF448559
JQ740789
MF448571
MF448558
MF448562
KC182545
NC008363
KX346236
KC182548
0.06
0.05
0.04
0.03
0.02
0.01
0.00

Figure S2

Phylogeny of OTUs from Norwegian cheese production and published 936 phage genome sequences and OTUs inferred from genome sequences of 936 phages. OTUs only found in dairy 1 is shown in green, OTUs only found in dairy 2 is shown in blue, OTUs found in both dairy plants are shown in pink. OTUs representing phages isolated in this work are shown in black. OTUs representing published phage genome sequences (66 uniue OTUs) are shown in grey. The published sequences are represented by their accession numbers.
