# Supplementary material for: Unprecedented Diversity of Lactococcal Group 936 Bacteriophages Revealed by Amplicon Sequencing of the Portal Protein Gene
Source: Viruses. 2019 May 16;11(5):443. doi: 10.3390/v11050443 (PMC6563314; doi:10.3390/v11050443)
Supplement: Supplementary file 1 [file viruses-11-00443-s001.zip › viruses-496482-proofread back suppl/supplementary tables.pdf]

Sample	Genome Size (bp)	Number of CDS
LMGA3	31,219	55
LMGA20	31,721	58
LMGA28	30,299	54
LMG803	28,890	53
LMG803J	30,328	53
LMG803V	29,738	54
LMG806	30,603	53
LMG807N	28,785	50
LMG807V	30,370	55
LMG812J	30,508	53
LMG815N	31,728	58
LMG824N	32,980	60
LMG825	30,730	55
LMG828	32,466	59
LMG840	28,522	54
LMG843	30,458	54
LMG844	30,188	55
LMG849	31,397	62

Table S1. Genomes of bacteriophages isolated in this study.

ORF	Product	Minimum Pairwise Identity	ORF Size (min-max)
ORF1	Terminase small subunit	94.1%	522-528
ORF2	Terminase large subunit	93.4%	1623-1629
ORF3	HNH endonuclease	95.0%	255-285
ORF4	portal protein	94.4%	1116-1167
ORF5	putative head protease	94.6%	501-576
ORF6	major capsid protein	94.6%	1068-1182
ORF7	putative structural protein	97.1%	264-264
ORF8	putative structural protein 2	93.8%	315-315
ORF9	structural protein 3	94.4%	294-342
ORF10	putative structural protein 4	92.9%	318-366
ORF11	major tail protein	88.6%	657-906
ORF12	putative structural protein 5	94.3%	276-276
ORF13	putative structural protein 6	96.4%	513-513
ORF14	tape measure protein	76.5%	2493-3120
ORF15	tail length tape measure protein	69.4%	747-1878
ORF18	receptor binding protein	71.5%	759-870
ORF19	holin	90.2%	276-354
ORF20	lysin	74.4%	681-786
ORF21	hypothetical protein	92.8%	261-279
ORF23	hypothetical protein	85.5%	189-348
ORF32	hypothetical protein	88.9%	384-534
ORF33	hypothetical protein	93.8%	213-246
ORF34	single-stranded DNA binding protein	89.6%	345-360
ORF38	hypothetical protein	87.0%	252-411
ORF39	hypothetical protein	90.7%	202-249
ORF41	hypothetical protein	90.4%	243-279
ORF52	holiday junction endonuclease	92.7%	132-132
ORF53	holiday junction endonuclease	91.9%	480-492
ORF54	middle expressed protein 4	92.1%	162-168

Table S2. Core genome of group 936 bacteriophages. The ORFs are number according to numbering in bacteriophage sk1.
